# Evaluation of the English Version of the Fear of COVID-19 Scale and Its Relationship with Behavior Change and Political Beliefs

**DOI:** 10.1007/s11469-020-00342-9

**Published:** 2020-06-15

**Authors:** Taylor Winter, Benjamin C. Riordan, Amir H. Pakpour, Mark D. Griffiths, Andre Mason, John W. Poulgrain, Damian Scarf

**Affiliations:** 1 Department of Psychology, Victoria University of Wellington, Wellington, New Zealand; 2 Discipline of Addiction Medicine, Central Clinical School, Faculty of Medicine and Health, University of Sydney, Sydney, Australia; 3 Department of Nursing, School of Health and Welfare, Jönköping University, Jönköping, Sweden; 4 International Gaming Research Unit, Psychology Department, Nottingham Trent University, Nottingham, UK; 5 Department of Psychology, University of Otago, Dunedin, New Zealand

**Keywords:** COVID-19, Fear, Psychometrics, Fear of COVID-19 scale, Behavior change

## Abstract

The COVID-19 pandemic has many individuals around the world fearing for their lives. The constant news coverage, rapid transmission, and relatively high mortality rate, make fearfulness a natural response. To assess the fear of COVID-19, the Fear of COVID-19 Scale (FCV-19S) was developed. The primary aim of the present study was to conduct the first psychometric assessment and validation of the English version of the FCV-19S. Two samples were collected in New Zealand. Sample 1 comprised 1624 participants of which 1397 completed all questions and were used in the analyses. Sample 2 comprised 1111 participants of which 1023 completed all questions and were used in the analyses. Several psychometric tests were conducted to ascertain the scale’s reliability and validity. Across both samples, the FCV-19S had high internal consistency. Consistent with the earlier validation studies, the FCV-19S displayed a moderately strong relationship with the perceived infectability and germ aversion subscales of the perceived vulnerability to disease scale (PVDS). Furthermore, FCV-19S scores were negatively correlated with the Warwick-Edinburgh Mental Wellbeing Scale (WEMWBS) scores. With respect to the motivating role of fear, there was a significant relationship between FCV-19S scores and adherence to the lockdown rules that were implemented in New Zealand. Finally, consistent with recent reports on the politicization of the COVID-19 pandemic, an exploratory question found that participants who rated themselves as more conservative tended to report lower FCV-19S scores. The English version of the COVID-19S is a sound unidimensional scale with robust psychometric properties and can be used with confidence among English-speaking populations.

**Electronic supplementary material:**

The online version of this article (10.1007/s11469-020-00342-9) contains supplementary material, which is available to authorized users.

At the time of writing (May 20, 2020) the total number of novel coronavirus-2019 (COVID-19) cases is over five million worldwide, with over 345,000 deaths (Worldometers 2020). Worldometers (2020) is just one of several websites offering close to real-time updates on COVID-19 cases, with country-specific data (i.e., the total number of cases, total number of deaths, and rates per million of population). Although many individuals may choose to avoid reading information from websites, the constant news coverage on COVID-19 makes such information almost impossible to avoid, providing individuals with little respite from the worldwide suffering.

With news coverage emphasizing the rapid transmission and relatively high mortality rate, fearfulness is a natural response (Lin 2020; Pappas et al. 2009). While fear may motivate people to abide by public health messages that aim to reduce the spread of COVID-19, such as spatial distancing and handwashing (Harper et al. 2020; Pakpour and Griffiths 2020; Tannenbaum et al. 2015), it may also lead to a number of psychological (e.g., anxiety) and psychosocial (e.g., prejudice) consequences (Holmes et al. 2020; Mamun and Griffiths 2020; Sibley et al. 2020). For example, news coverage that the virus originated in China has resulted in Asian people in Western countries experiencing increased levels of racism (Aratani 2020; Biddle 2020; Jan 2020).

The first step in investigating the relationship between fear of COVID-19, and its association with psychological factors, psychosocial factors, and behavior change, is the development of a valid psychometric instrument. Consequently, Ahorsu et al. (2020) recently developed the Fear of COVID-19 Scale (FCV-19S). The FCV-19S assesses participant’s agreement with seven items (e.g., “I cannot sleep because I am worried about getting coronavirus-19”). The initial reliability and validity of the FCV-19S was conducted in Persian, with Ahorsu et al. (2020) reporting very good internal consistency (*α* = .82) and concurrent validity, with the scale positively correlating (*r* = .425) with a measure of anxiety and depression. In rapid time, the FCV-19S has been translated and validated in Bangla (Sakib et al. 2020), Italian (Soraci et al. 2020), Arabic (Alyami et al. 2020), Russian (Reznik et al. 2020), Hebrew (Bitan et al. 2020), and Turkish (Satici et al. 2020). The present paper presents the first psychometric assessment and validation of the scale in English.

## Methods

### Participants and Procedure

Two samples were collected in New Zealand. Sample 1 comprised 1624 participants of which 1397 completed all questions and were used in the analyses. Sample 1 was collected during Alert Level 4. Alert Level 4 was the highest level of lockdown imposed in New Zealand, with people instructed to stay at home and limit contact to the people that they live with (see *Lockdown Behavior* below for more detail on Alert Level 4). Sample 1 comprised more males (60.3%) than female participants, who were aged between 18 and 88 years old (mean [M] = 47.5 years; standard deviation [SD] = 16.3 years), and were largely New Zealand European (84.6%; 5.7% Maori or Pasifika, 1.8% Asian, and 7.9% other). Sample 2 comprised 1111 participants of which 1023 completed all questions and were used in the analyses. Sample 2 was collected during Alert Level 3. Level 3 immediately followed Alert Level 4, allowing people to expand their social circle to close family that they do not currently live with (see *Lockdown Behavior* below for more detail on Alert Level 3). Sample 2 comprised more female (69.7%) than male participants, who were aged between 18 and 85 years (M = 42.0 years; SD = 13.3 years), and were largely New Zealand European (75.4%; 7.6% Maori or Pasifika, 3.4% Asian, and 13.6% other).

With respect to recruitment, both samples were recruited via posts on popular social networking sites and articles via several New Zealand news websites. Participants who agreed to take part in the study clicked on a hyperlink and were taken to the study webpage. The first page included a description of the study and an electronic consent form. Participants who provided informed consent were asked to complete a 15-min survey. The study was reviewed and approved by the University of Otago Human Ethics Committee.

### Measures

#### Demographic Information

Age, gender (male, female, other), and ethnicity were collected.

#### Fear of COVID-19 Scale (FCV-19S)

The FCV-19S is a seven-item measure assessing the extent to which a person fears COVID-19 (Ahorsu et al. 2020). The scale asks participants to indicate the extent to which they agree with each item (e.g., “When I watch news and stories about coronavirus 2019 on social media, I become nervous or anxious”) from 1 (*strongly disagree*) to 7 (*strongly agree*). Both Sample 1 (*α* = .89) and Sample 2 (*α* = .88) completed the FCV-19S.

#### Perceived Vulnerability to Disease Scale

The Perceived Vulnerability to Disease Scale (PVDS) is a 15-item measure of an individual’s perceived vulnerability to disease and asks them to rate from 1 (*strongly disagree*) to 7 (*strongly agree*) the extent to which they agree with each item (Duncan et al. 2009). The scale has two subscales. Seven items assess perceived infectability (e.g., “I have a history of susceptibility to infectious diseases”) and eight items assess germ aversion (e.g., “It really bothers me when people sneeze without covering their mouths”). Both Sample 1 (perceived infectability *α* = 0.90, germ aversion *α* = 0.71) and Sample 2 (perceived infectability *α* = 0.92, germ aversion *α* = 0.77) completed the PVDS. See Supplementary Table 1 for confirmatory factor analysis.

#### Warwick-Edinburgh Mental Wellbeing Scale

The Warwick-Edinburgh Mental Wellbeing Scale (WEMWBS) is a 14-item measure of mental wellbeing. Participants are asked to indicate from 1 (*none of the time*) to 5 (*all of the time*) how often they experienced each statement (e.g., “I’ve been feeling optimistic about the future”, “I’ve been feeling close to other people”) (Tennant et al. 2007). Only Sample 2 (*α* = .91) completed the WEMWBS. See Supplementary Table 2 for confirmatory factor analysis.

#### Lockdown Behavior

A State of National Emergency was declared in New Zealand on March 25, 2020, providing the New Zealand Government with access to “…extra-ordinary powers that will support delivery of an effective and timely response to COVID-19” (Ardern 2020). In brief, this allowed the implementation of a four-level Alert System. Alert Level 4 was implemented between March 26 and April 27, with all businesses (expect essential services) and educational facilities closed. Moreover, a set of rules regarding personal movement were implemented, with people instructed to stay home, only associate with individuals they live with, and limit travel to their local area. Alert Level 3 immediately followed Alert Level 4, implemented between April 28 and May 3. Alert Level 3 allowed some businesses and educational facilities to open. However, social contact was still severely limited, with individuals only allowed to extend their social circle to close family they do not currently live with. To assess whether participants abided by New Zealand’s lockdown rules, participants were asked to indicate from 1 (*strongly disagree*) to 7 (*strongly agree*) whether they abided by the five rules (e.g., “I ensure I maintain the 2-meter rule when out in public”) (Table 3).

#### Political Beliefs

Given recent work suggesting COVID-19 has become highly politicized in the USA (Conway et al. 2020; Rothgerber et al. 2020), the survey also included a single exploratory item to assess participants’ political beliefs (Jost 2006; Sibley and Wilson 2007). More specifically, following Sibley and Wilson (2007), participants were given the instructions “Often, people use the terms ‘liberal’ or ‘conservative’ to describe their political beliefs. How would you rate yourself in these terms?” (p. 75). Participants responded on a scale ranging from 1 (*very liberal*), through 4 (*moderate*), to 7 (*very conservative*).

### Data Analysis

Descriptive statistics were used to describe the study participants’ characteristics. The analysis also tested the skewness, kurtosis, and distributions of each scale item in the FCV-19S, the internal consistency (Cronbach’s alpha), and inter- and item-total correlations. A confirmatory factor analysis (CFA) was conducted using maximum likelihood estimation to measure the factor structure of the FCV-19S and report the factor loadings and the goodness of fit using root mean square error of approximation (RMSEA where good fit is typically less than 0.1) and comparative fit index (CFI where good fit is typically more than 0.9).

To date, most published psychometric reports have focused on classical test theory. The use of classical test theory, in which raw scores, linear combinations of these scores, and responses that are ordinal in scale, is considered as data on an interval scale. Rasch analysis is a statistical technique traditionally for binary data, but some polytomous generalizations can also be used for interval data (Lin et al. 2017). Standard Rasch analysis is based on unidimensional models. In unidimensional models, it is assumed that only one hidden feature of the individual determines the individual’s performance in the scale. If the data do not fit well with the Rasch model, the unidimensional assumption is rejected. This means that more than one hidden feature has affected an individual’s performance, so the feature cannot be assessed well using the scale in question (Lin and Pakpour 2017).

Here, Rasch analysis using the partial credit model was used to assess the unidimensionality and item fits of the FCV-19S (Masters 1982). Item validity was assessed using information-weighted fit statistic (infit) mean square (MnSq), and outlier-sensitive fit statistic (outfit) MnSq with values between 0.5 and 1.5 considered acceptable. The presence of disordering threshold in the FCV-19S was assessed using average and step measures of the descriptors. A monotonic increase in difficulties between 0.5 and 1.5 suggests no disordering. The unidimensionality of the FCV-19S was examined by conducting principal component analysis of the residuals (PCAR) on the items. Explaining at least 50% of the variance in the Rasch dimension, and an eigenvalue of less than 2.0 on first contrast, provides evidence of unidimensionality (Linacre 2012). The response pattern across subgroups of the population (age and gender groups) was assessed by differential item functioning (DIF). All analyses were conducted in R (version 4.0.0; R Core Team, 2019) using the LAVAAN (Rosseel 2012) and WINSTEPS 3.71 software (Linacre 2012).

To test the concurrent validity, Pearson’s correlations were used to assess the relationship between the FCV-19S and the PVDS (Samples 1 and 2), the WEMWBS (Sample 2), and adherence to lockdown rules (Samples 1 and 2). Finally, Spearman rank order correlations were used to test the relationship between FCV-19S and political beliefs.

## Results

For Samples 1 and 2 respectively, the mean FCV-19S score was 15.6 and 18.3 (SD = 7.7 and 7.9), PVD was 57.0 and 58.5 (SD = 10.9 and 14.0), and WEMWBS was 49.1 (SD = 8.7). Table 1 shows the summary statistics and item analysis for each scale item in the FCV-19S (mean item scores, standard deviation, skew, kurtosis, item difficulty, item discrimination, and internal consistency if item was deleted). As seen in Table 1, items 3, 6, and 7 were not normally distributed (indicated by a Skew and Kurtosis falling outside of the − 2 to 2 range), due to the fact that most participants “strongly disagreed” with these items. Finally, the internal consistency if an item was deleted ranged from .84 to .86, suggesting that dropping any item in the scale would not improve overall internal consistency.

**Table 1:** Summary statistics and item analysis for each scale item in the FCV-19S

	Question	Mean	SD	Skew	Kurtosis	α if Del.
S1	I am most afraid of coronavirus-19.	3.06	1.71	0.47	− 1.02	0.863
	It makes me uncomfortable to think about coronavirus-19.	2.88	1.7	0.65	− 0.81	0.853
	My hands become clammy when I think about coronavirus-19.	1.57	1.01	2.52	7.56	0.86
	I am afraid of losing my life because of coronavirus-19.	2.16	1.53	1.37	0.93	0.86
	When watching news and stories about coronavirus-19 on social media, I become nervous or anxious.	2.66	1.73	0.77	−0.69	0.857
	I cannot sleep because I am worrying about getting coronavirus-19.	1.65	1.14	2.33	5.58	0.859
	My heart races or palpitates when I think about getting coronavirus-19.	1.61	1.13	2.38	5.69	0.856
S2	I am most afraid of coronavirus-19.	3.62	1.68	0.04	− 1.2	0.855
	It makes me uncomfortable to think about coronavirus-19.	3.45	1.76	0.18	− 1.28	0.845
	My hands become clammy when I think about coronavirus-19.	1.73	1.02	1.9	4.22	0.856
	I am afraid of losing my life because of coronavirus-19.	2.42	1.57	1.06	0.09	0.856
	When watching news and stories about coronavirus-19 on social media, I become nervous or anxious.	3.3	1.77	0.18	− 1.29	0.855
	I cannot sleep because I am worrying about getting coronavirus-19.	1.89	1.24	1.75	2.88	0.853
	My heart races or palpitates when I think about getting coronavirus-19.	1.87	1.28	1.72	2.38	0.851

CFA of the FCVS-19 scale showed good fit with CFI of 0.90 for Sample 1 and 0.92 for Sample 2 (Table 2). RMSEA was slightly higher than levels indicative of good fit, with RMSEA of 0.16 for Sample 1 and 0.13 for Sample 2, where a traditional cutoff of good fit is normally less than 0.1. Both samples, however, independently support a similar level of fit and all significantly loaded onto the same latent factor with standardized loadings between 0.6 and 0.8.

**Table 2:** CFA for the FCV-19S

	FCV-19S item	Estimate	Std. Err	z-value	*p*(> |z|)
S1	1	0.64			
	2	0.72	0.05	22.67	< 0.001
	3	0.78	0.03	24.23	< 0.001
	4	0.67	0.04	21.38	< 0.001
	5	0.72	0.05	22.79	< 0.001
	6	0.77	0.03	23.86	< 0.001
	7	0.80	0.03	24.75	< 0.001
S2	1	0.67			
	2	0.75	0.06	20.86	< 0.001
	3	0.75	0.03	20.69	< 0.001
	4	0.67	0.05	18.76	< 0.001
	5	0.70	0.06	19.61	< 0.001
	6	0.73	0.04	20.35	< 0.001
	7	0.75	0.04	20.74	< 0.001

All items fitted well with their latent construct as the infit and outfit MNSQ were within acceptable range (0.5–1.5) in both samples (Table 3). The most difficult item for Sample 1 and Sample 2 was “my hands become clammy when I think about coronavirus-19”*.* In contrast, the easiest item for Sample 1 and Sample 2 was “I am most afraid of coronavirus-19”*.* A monotonic increase in threshold values (i.e., average and step measures) for item difficulties (i.e., the seven-point Likert scales) was found in both samples. No DIF was seen across gender and age subgroups of both Sample 1 and Sample 2 (i.e., DIF contrasts < 0.5). The Rasch dimension demonstrated that the FCV-19S explained 64.2% and 67.2% of the variance in Sample 1 and Sample 2, with eigenvalues of 12.57 and 14.32, respectively. The first contrasts gave eigenvalues of 1.84 and 1.74 for both Sample 1 and Sample 2, respectively.

**Table 3:** Analyses from Rasch model for the FCV-19S

	Question	Infit MnSq	Outfit MnSq	Difficulty	Discrimination	DIF contrast across gender^cd^	DIF contrast across age^ce^
S1	I am most afraid of coronavirus-19.	1.11	1.17	− 1.04	0.83	− 0.06	0.32
	It makes me uncomfortable to think about coronavirus-19.	0.94	0.98	− 0.86	1.03	0.06	0.10
	My hands become clammy when I think about coronavirus-19.	0.87	0.72	0.96	1.13	− 0.12	− 0.11
	I am afraid of losing my life because of coronavirus-19.	1.36	1.25	− 0.06	0.87	− 0.25	0.43
	When watching news and stories about coronavirus-19 on social media, I become nervous or anxious.	1.13	1.12	− 0.64	0.98	0.18	− 0.49
	I cannot sleep because I am worrying about getting coronavirus-19.	1.07	0.92	0.78	1.09	− 0.02	− 0.19
	My heart races or palpitates when I think about getting coronavirus-19.	1.00	0.80	0.87	1.15	0.22	−0.36
S2	I am most afraid of coronavirus-19.	1.11	1.12	− 1.11	0.85	−0.04	0.32
	It makes me uncomfortable to think about coronavirus-19.	0.93	0.91	− 0.95	1.12	0.05	0.00
	My hands become clammy when I think about coronavirus-19.	0.84	0.69	1.11	1.15	0.00	− 0.08
	I am afraid of losing my life because of coronavirus-19.	1.30	1.29	0.10	0.82	− 0.19	0.42
	When watching news and stories about coronavirus-19 on social media, I become nervous or anxious.	1.16	1.19	− 0.81	0.92	0.10	− 0.43
	I cannot sleep because I am worrying about getting coronavirus-19.	0.97	0.87	0.82	1.09	0.00	− 0.08
	My heart races or palpitates when I think about getting coronavirus-19.	1.06	0.89	0.85	1.14	0.04	− 0.24

^c^DIF contrast > 0.5 indicates substantial DIF

^d^DIF contrast across gender: difficulty for males-difficulty for females

^e^DIF contrast across age: difficulty for participants with younger age (i.e., ≤ years)-difficulty for participants with older ages (i.e., ≥)

*MnSq* mean square error, *DIF* differential item functioning

To assess concurrent validity, the FCV-19S was correlated with perceived vulnerability to disease (Sample 1 and 2) and wellbeing (Sample 2). The FCV-19S demonstrated good concurrent validity. More specifically, there was a moderately strong relationship between the FCV-19S and the two subscales of perceived vulnerability to disease scale: perceived infectability (Sample 1: *r* = 0.35, *p* < 0.001; Sample 2: *r* = 0.40, *p* < 0.001) and germ aversion (Sample 1: *r* = 0.39, *p* < 0.001; Sample 2: *r* = 0.45, *p* < 0.001). In addition, there was a negative relationship between FCV-19S and the WEMWBS (*r* = −0.31, *p* < 0.001), such that those who had higher fear scores reported lower overall wellbeing scores. Finally, when assessing participants’ adherence to New Zealand’s Lockdown Rules, FCV-19S was significantly associated with adherence with all five rules during Alert Level 4 (Sample 1) and three of the five rules during Alert Level 3 (Sample 2) (Table 4).

**Table 4:** Means (M) and standard deviations (SD) for adherence to the lockdown rules

Lockdown rule item	*M*	*SD*	*rho*	*p*
I ensure I maintain the 2-m rule when out in public.	5.61	1.21	0.20	< 0.001
I keep my physical activity local, limiting it to an outdoor place that can be readily accessed on foot from my home.	5.78	1.35	0.17	< 0.001
I only drive for essential reasons, such as going to work or picking up essential supplies from the supermarket.	5.87	1.31	0.14	< 0.001
When going to the supermarket, we only send one member of our household.	5.85	1.43	0.08	< 0.001
I have not been within 2-m of family or friends outside of my bubble.	5.65	1.63	0.12	< 0.001
I ensure I maintain the 2-m rule when out in public.	6.23	0.98	0.11	< 0.001
I keep my physical activity local, limiting it to an outdoor place that can be readily accessed on foot from my home.	6.61	0.866	0.14	< 0.001
I only drive for essential reasons, such as going to work or picking up essential supplies from the supermarket.	6.54	0.956	0.05	0.13
When going to the supermarket, we only send one member of our household.	6.3	1.42	0.02	0.56
I have not been within 2-m of family or friends outside of my bubble.	6.4	1.09	0.12	< 0.001

Also shown is the association between FCV-19S and adherence to each rule

Finally, consistent with the view that COVID-19 has become a politicized topic, there were modest negative correlations between the FCV-19S scores and political beliefs (Sample 1: *M* = 3.74, *SD* = 1.49, *rho* = −.20, *p* < .001; Sample 2: *M* = 2.57, *SD* = 1.15, *rho* = −.07, *p* = .014). That is, participants that rated themselves as more toward the conservative end of the political spectrum tended to report lower FCV-19S scores.

## Discussion

The primary aim of the present study was to evaluate the English version of the FCV-19S. Across two large samples, the FCV-19S had high internal consistency. Mirroring the Bangla (Sakib et al. 2020), Italian (Soraci et al. 2020), Arabic (Alyami et al. 2020), and Hebrew (Bitan et al. 2020) validation studies, participants tended to “strongly disagree” with items 3, 6, and 7. As Alyami et al. (2020) note, items 3, 6, and 7 refer to somatic responses to COVID-19 fear (e.g., “My heart races or palpitates when I think about getting coronavirus-19”), rather than the more general level of fear tapped by the remaining items (e.g., “When watching news and stories about coronavirus-19 on social media, I become nervous or anxious”). Consistent with the earlier validation studies, the FCV-19S displayed a moderately strong relationship with the perceived infectability and germ aversion subscales of the PVD scale (Duncan et al. 2009). Moreover, the FCV-19S scores were negatively correlated with the WEMWBS, reflecting the fact that as fear of COVID-19 increased wellbeing decreased. This finding is consistent with previous studies reporting positive correlations between the FCV-19S and the hospital anxiety and depression scale (Ahorsu et al. 2020; Alyami et al. 2020), Patient Health Questionnaire (Sakib et al. 2020), and the depression and anxiety stress scale (Bitan et al. 2020).

With respect to the motivating role of fear, there was a significant relationship between FCV-19S scores and adherence to the lockdown rules that were implemented in New Zealand (Harper et al. 2020; Pakpour and Griffiths 2020; Tannenbaum et al. 2015). For example, fear of COVID-19 was associated with participants maintaining the 2-m rule (i.e., maintain 2-m distance from other people when out in public) and keeping physical activity to outdoor places that could be readily accessed by foot. This finding is consistent with Harper et al. (2020), who reported a positive correlation between FCV-19S scores and participants judgment of the degree to which several behaviors and practices had changed due to the pandemic (e.g., hand washing, care of children, and the elderly).

Consistent with the view that the politicization of COVID-19 is not limited to the USA, participants that rated themselves as more conservative displayed lower FCV-19S scores than participants that rated themselves more toward the liberal end of the political spectrum. Although conservative politicians in New Zealand have not gone as far as USA President Donald Trump, who early on referred to the virus as a hoax (Franck 2020), they have argued that the liberal government should reduce alert levels more quickly, effectively communicating a lower level of risk (Burrows 2020; Coughlan 2020). Indeed, in the USA, Rothgerber et al. (2020) demonstrated that perceptions of health risk (e.g., the likelihood of being infected with COVID-19) mediated the relationship between conservatism and abiding by social distancing rules.

Although the study comprised over 3000 participants in total, there are some limitations to consider when interpreting the results. Data were not collected from a nationally representative sample (i.e., they comprised a self-selecting sample) and all the data were self-report which is subject to established method biases. Given the cross-sectional nature of the data, the relationships between all the variables examined are associational, and the only way to establish the directional nature of the variables would be to carry out a longitudinal study. However, for a scale validation study, the design was robust.

## Conclusion

The current study demonstrates that the English version of the COVID-19S is a sound unidimensional scale with robust psychometric properties that can be used with confidence among English-speaking populations. Critically, the COVID-19S displays sound concurrent validity and is associated with adherence to public health messages that aim to reduce the spread of COVID-19 (e.g., maintaining the 2-m rule).

## Electronic supplementary material

ESM 1 (DOCX 19 kb).
