# Supplementary material for: Evaluation of the English Version of the Fear of COVID-19 Scale and Its Relationship with Behavior Change and Political Beliefs
Source: Int J Ment Health Addict. 2020 Jun 15;21(1):372–82. doi: 10.1007/s11469-020-00342-9 (PMC7295324; doi:10.1007/s11469-020-00342-9)
Supplement: Supplementary file 1 — (DOCX 19 kb). [file 11469_2020_342_MOESM1_ESM.docx]

Supplementary Table 1. CFA for PVDS

|  | Subscale | PVD Item | Estimate | Std.Err | z-value | P(>\|z\|) | Std.lv | Std.all |
| --- | --- | --- | --- | --- | --- | --- | --- | --- |
| Sample 1 | Perceived infectability | 2 | 1 |  |  |  | 0.849 | 0.67 |
|  |  | 5 | 0.924 | 0.044 | 20.795 | < 0.01 | 0.785 | 0.607 |
|  |  | 6 | 1.314 | 0.049 | 26.995 | < 0.01 | 1.116 | 0.817 |
|  |  | 8 | 1.472 | 0.051 | 28.997 | < 0.01 | 1.25 | 0.895 |
|  |  | 10 | 1.27 | 0.046 | 27.364 | < 0.01 | 1.079 | 0.831 |
|  |  | 12 | 1.03 | 0.043 | 23.828 | < 0.01 | 0.875 | 0.707 |
|  |  | 14 | 0.967 | 0.043 | 22.662 | < 0.01 | 0.821 | 0.668 |
|  | Germ aversion | 1 | 1 |  |  |  | 0.555 | 0.421 |
|  |  | 3 | 1.166 | 0.111 | 10.538 | < 0.01 | 0.648 | 0.431 |
|  |  | 4 | 1.15 | 0.111 | 10.36 | < 0.01 | 0.639 | 0.418 |
|  |  | 7 | 1.708 | 0.133 | 12.863 | < 0.01 | 0.948 | 0.685 |
|  |  | 9 | 1.125 | 0.115 | 9.809 | < 0.01 | 0.625 | 0.382 |
|  |  | 11 | 1.302 | 0.114 | 11.465 | < 0.01 | 0.723 | 0.506 |
|  |  | 13 | 1.094 | 0.099 | 11.018 | < 0.01 | 0.607 | 0.467 |
|  |  | 15 | 1.398 | 0.114 | 12.296 | < 0.01 | 0.776 | 0.595 |
| Sample 2 | Perceived infectability | 2 | 1 |  |  |  | 1.112 | 0.746 |
|  |  | 5 | 1.005 | 0.044 | 22.733 | < 0.01 | 1.118 | 0.704 |
|  |  | 6 | 1.253 | 0.045 | 28.002 | < 0.01 | 1.394 | 0.85 |
|  |  | 8 | 1.323 | 0.045 | 29.436 | < 0.01 | 1.471 | 0.889 |
|  |  | 10 | 1.133 | 0.042 | 27.23 | < 0.01 | 1.26 | 0.829 |
|  |  | 12 | 1.007 | 0.043 | 23.662 | < 0.01 | 1.12 | 0.73 |
|  |  | 14 | 0.94 | 0.041 | 22.967 | < 0.01 | 1.045 | 0.711 |
|  | Germ aversion | 1 | 1 |  |  |  | 0.598 | 0.47 |
|  |  | 3 | 1.567 | 0.141 | 11.153 | < 0.01 | 0.937 | 0.514 |
|  |  | 4 | 1.614 | 0.14 | 11.514 | < 0.01 | 0.965 | 0.545 |
|  |  | 7 | 2.085 | 0.161 | 12.97 | < 0.01 | 1.247 | 0.707 |
|  |  | 9 | 1.002 | 0.112 | 8.921 | < 0.01 | 0.599 | 0.366 |
|  |  | 11 | 1.656 | 0.141 | 11.764 | < 0.01 | 0.99 | 0.567 |
|  |  | 13 | 1.564 | 0.133 | 11.775 | < 0.01 | 0.935 | 0.568 |
|  |  | 15 | 1.537 | 0.126 | 12.182 | < 0.01 | 0.919 | 0.609 |

Supplementary Table 2. CFA for WEMWBS

| WEMWBS Item | Estimate | Std.Err | z-value | P(>\|z\|) | Std.lv | Std.all |
| --- | --- | --- | --- | --- | --- | --- |
| 1 | 1 |  |  |  | 0.511 | 0.602 |
| 2 | 1.003 | 0.064 | 15.647 | < 0.01 | 0.512 | 0.539 |
| 3 | 1.14 | 0.064 | 17.936 | < 0.01 | 0.583 | 0.642 |
| 4 | 0.799 | 0.058 | 13.743 | < 0.01 | 0.408 | 0.462 |
| 5 | 1.199 | 0.072 | 16.715 | < 0.01 | 0.613 | 0.586 |
| 6 | 1.15 | 0.057 | 20.052 | < 0.01 | 0.588 | 0.748 |
| 7 | 1.129 | 0.058 | 19.352 | < 0.01 | 0.577 | 0.711 |
| 8 | 1.469 | 0.068 | 21.718 | < 0.01 | 0.751 | 0.843 |
| 9 | 1.201 | 0.069 | 17.388 | < 0.01 | 0.614 | 0.616 |
| 10 | 1.503 | 0.069 | 21.669 | < 0.01 | 0.768 | 0.84 |
| 11 | 0.883 | 0.054 | 16.224 | < 0.01 | 0.451 | 0.564 |
| 12 | 1.037 | 0.064 | 16.298 | < 0.01 | 0.53 | 0.567 |
| 13 | 1.245 | 0.069 | 18.006 | < 0.01 | 0.636 | 0.645 |
| 14 | 1.398 | 0.066 | 21.242 | < 0.01 | 0.714 | 0.815 |
